# Supplementary material for: Circulating Let-7 Family Members as Non-Invasive Biomarkers for Predicting Hepatocellular Carcinoma Risk after Antiviral Treatment among Chronic Hepatitis C Patients
Source: Cancers (Basel). 2022 Apr 16;14(8):2023. doi: 10.3390/cancers14082023 (PMC9030777; doi:10.3390/cancers14082023)
Supplement: Supplementary file 1 [file cancers-14-02023-s001.zip › cancers-1648230-supplementary.pdf]

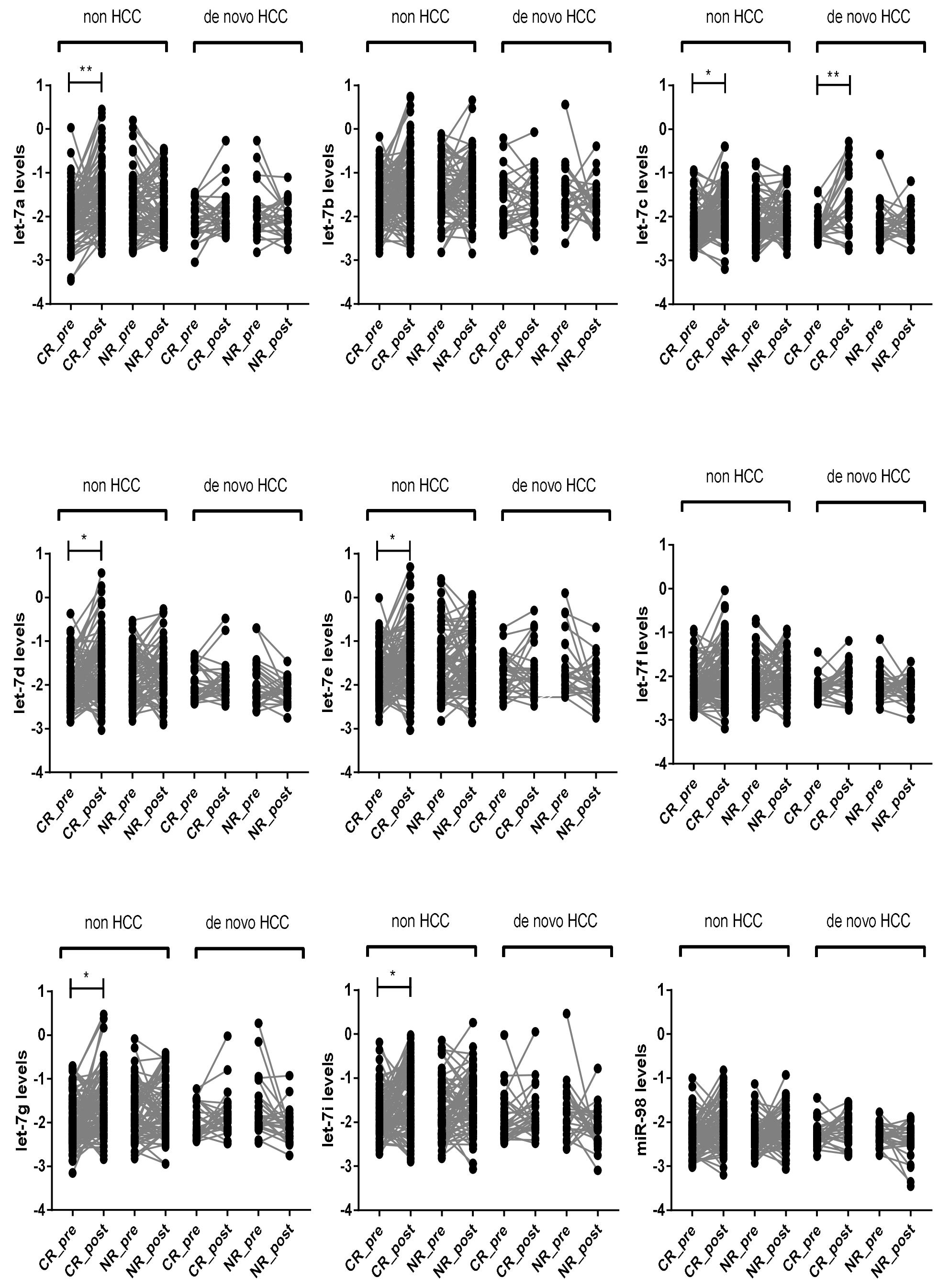

Figure S1. Scatter plots comparing the pre-treatment (baseline) and post-treatment (M6) circulating Let-7 family expression between SVR and non-SVR groups among the de novo HCC and non-HCC CHC patients. Let-7 levels based on Log $_{10}2^{-\Delta Ct}$ ($Ct_{target} - Ct_{cel39}$) method. The *p-value* was represented by Dunn's multiple comparisons test.

(A)

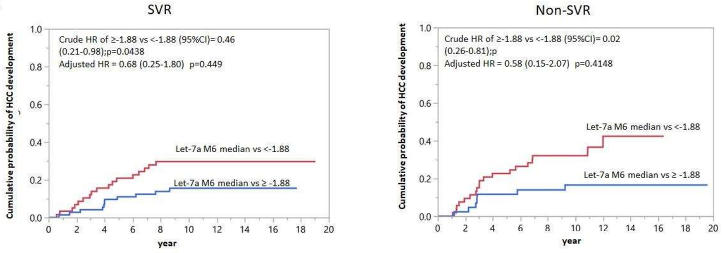

SVR		2	4	6	8	10	12	14	16
Let-7a Median≥-1.88	No. at risk	72	68	66	59	24	14	8	2
	Cumulative incidence	2.74%	8.22%	10.96%	13.79%	15.51%	15.51%	15.51%	15.51%
Let-7a median<-1.88	No. at risk	54	49	45	41	25	21	17	8
	Cumulative incidence	7.02%	15.79%	22.81%	29.82%	29.82%	29.82%	29.82%	29.82%

Non-SVR		2	4	6	8	10	12	14	16
Let-7a median≥-1.88	No. at risk	43	39	38	38	29	9	4	2
	Cumulative incidence	2.30%	11.63%	13.95%	13.95%	16.56%	16.56%	16.56%	16.56%
Let-7a median<-1.88	No. at risk	49	42	40	36	22	11	9	3
	Cumulative incidence	9.43%	22.64%	26.42%	32.08%	32.08%	42.37%	42.37%	42.37%

(B)

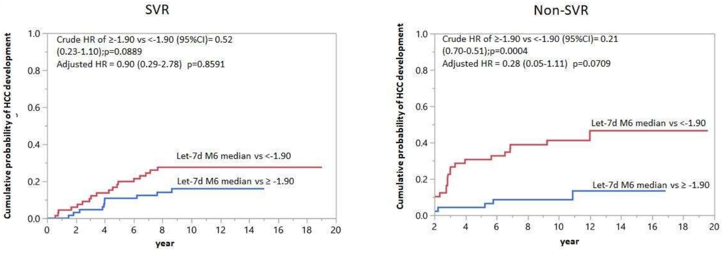

SVR		2	4	6	8	10	12	14	16
Let-7d Median≥-1.90	No. at risk	64	60	59	52	20	10	7	1
	Cumulative incidence	3.08%	9.23%	10.77%	13.96%	15.92%	15.92%	15.92%	15.92%
Let-7d median<-1.90	No. at risk	62	57	52	48	29	25	21	9
	Cumulative incidence	6.15%	13.85%	21.54%	27.69%	27.69%	27.69%	27.69%	27.69%

Non-SVR		2	4	6	8	10	12	14	16
Let-7d median≥-1.90	No. at risk	47	46	44	43	30	9	4	2
	Cumulative incidence	2.13%	4.26%	8.51%	8.51%	8.51%	13.33%	13.33%	13.33%
Let-7d median<-1.90	No. at risk	45	35	34	31	21	11	9	4
	Cumulative incidence	10.20%	30.61%	32.65%	38.78%	41.13%	46.48%	46.48%	46.48%

(C)

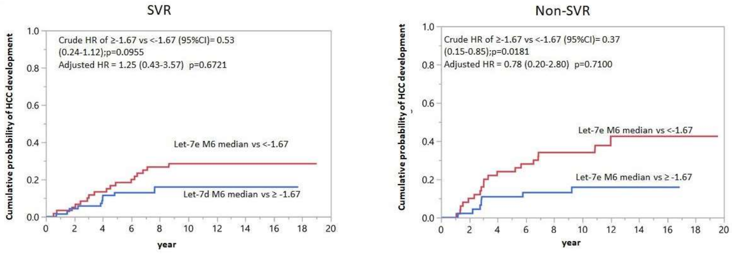

SVR		2	4	6	8	10	12	14	16
Let-7e Median≥-1.67	No. at risk	68	64	62	55	21	11	8	2
	Cumulative incidence	4.29%	10.00%	12.86%	15.91%	15.91%	15.91%	15.91%	15.91%
Let-7e median<-1.67	No. at risk	58	53	49	45	28	24	20	8
	Cumulative incidence	5.00%	13.33%	20.00%	26.67%	28.46%	28.46%	28.46%	28.46%

Non-SVR		2	4	6	8	10	12	14	16
Let-7e median≥-1.67	No. at risk	45	42	41	40	26	7	2	2
	Cumulative incidence	4.35%	10.87%	13.04%	13.04%	15.85%	15.85%	15.85%	15.85%
Let-7e median<-1.67	No. at risk	46	39	37	30	25	13	11	4
	Cumulative incidence	10.00%	24.00%	28.00%	34.00%	34.00%	42.46%	42.46%	42.46%

(D)

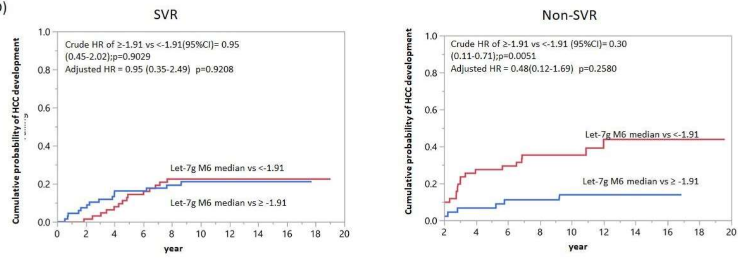

SVR		2	4	6	8	10	12	14	16
Let-7g Median≥-1.91	No. at risk	64	59	58	53	18	9	6	2
	Cumulative incidence	7.35%	14.71%	16.18%	19.20%	21.04%	21.04%	21.04%	21.04%
Let-7f median<-1.91	No. at risk	62	58	53	47	31	26	22	8
	Cumulative incidence	1.61%	8.06%	16.13%	22.61%	22.61%	22.61%	22.61%	22.61%

Non-SVR		2	4	6	8	10	12	14	16
Let-7g median≥-1.91	No. at risk	45	43	41	40	28	7	3	2
	Cumulative incidence	2.30%	11.63%	13.95%	13.95%	16.56%	16.56%	16.56%	16.56%
Let-7g median<-1.91	No. at risk	47	38	37	34	23	13	10	4
	Cumulative incidence	9.80%	27.45%	31.37%	35.29%	35.29%	43.78%	43.78%	43.78%

Figure S2. Kaplan-Meier survival analysis of let-7a, let-7d, let-7e, and let-7g (belong to Cluster 1) with HCC development in SVR and non-SVR group. Comparison of the cumulative probability of HCC development divided cut-off value from the median value of the distribution of Let-7 expression based on Log10 2^−delta Ct method in (**A**) let-7a, (**B**) let-7d, (**C**) let-7e, and (**D**) let-7g. HR: Hazard ratio; CI: Confidence interval; HCC: Hepatocellular carcinoma.

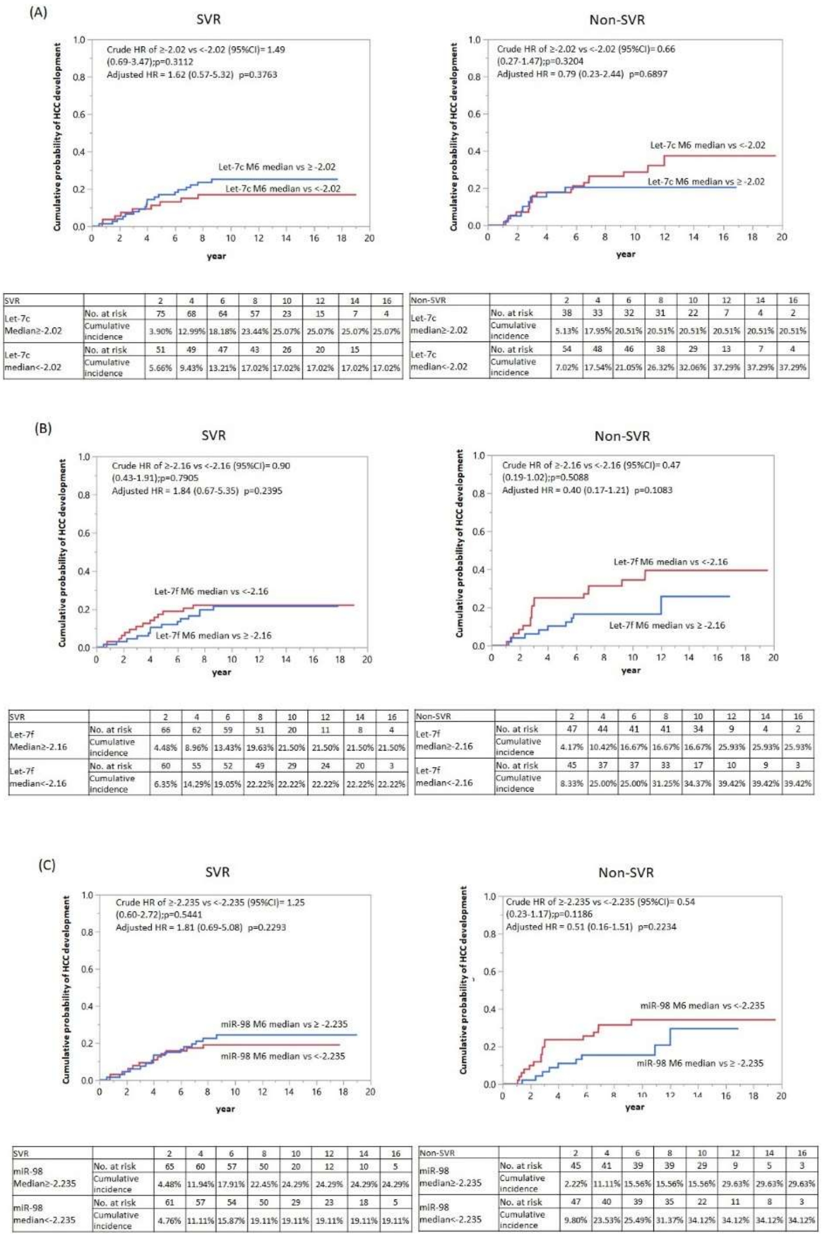

Figure S3. Kaplan-Meier survival analysis of let-7c, let-7f, and miR-98 (belong to Cluster 3) with HCC development in the SVR and non-SVR groups. Comparison of the cumulative probability of HCC development divided cut-off value from the median value of the distribution of Let-7 expression based on Log10 2^-delta Ct method in (**A**) let-7c (**B**) let-7f, and (**C**) miR-98. HR: Hazard ratio; CI: Confidence interval; HCC: Hepatocellular carcinoma.

Table S1. De novo HCC patients (n = 54) characteristics by BCLC stage.

BCLC Stage	n	%	Age(yr) at HCC	*p-Value*	Censored yr (Post M6 to HCC	*p-Value*	Tumor Size (cm)	*p-Value*
0	14	25.93	60.10 ± 6.45	rf	3.37 ± 2.25	rf	1.55 ± 0.19	rf
A	23	42.59	61.05 ± 8.67	ns	3.41 ± 2.00	ns	2.76 ± 0.83	<0.0001
B	5	9.26	60.43 ± 13.26	ns	5.64 ± 3.91	ns	3.44 ± 1.12	0.0015
C	12	22.22	67.89 ± 9.46	0.0253	5.81 ± 2.83	0.0288	4.82 ± 3.85	0.0493

Data are presented as the mean ± SD. Statistical analysis was performed using Nonparametric multiple tests (BCLC stage 0 as reference). Tumor size was measured in centimeters (cm) from the largest diameter of the largest tumor. BCLC: Barcelona clinic liver cancer.

Table S2. Correlation coefficients were calculated for the let-7 family and the FIB-4 score of post-anti-viral treatment was stratified by a FIB-4 score of 3.25(M6).

Log 10 2^–Delta Ct (M6)	FIB-4 Score <3.25				FIB-4 Score ≥3.25			
	Correlation Coefficient	Lower 95%	Upper 95%	*p*	Correlation Coefficient	Lower 95%	Upper 95%	*p*
let7a	−0.2609	−0.4139	−0.0935	0.0026	−0.1754	−0.4326	0.1083	0.2232
let7b	−0.2116	−0.3697	−0.0416	0.0153	−0.0983	−0.3666	0.1851	0.4971
let7c	−0.1415	−0.3056	0.0308	0.1069	−0.0379	−0.3129	0.243	0.794
let7d	−0.3353	−0.4793	−0.1738	<.0001	−0.0974	−0.3658	0.186	0.501
let7e	−0.2592	−0.4124	−0.0918	0.0028	−0.1721	−0.4298	0.1116	0.2321
let7f	−0.2296	−0.3859	−0.0605	0.0083	−0.1149	−0.3811	0.1689	0.4269
let7g	−0.2085	−0.3669	−0.0383	0.0169	−0.1654	−0.4242	0.1184	0.2509
let7i	−0.2181	−0.3756	−0.0484	0.0123	−0.1794	−0.436	0.1042	0.2126
miR98	−0.2742	−0.4257	−0.1077	0.0015	−0.0941	−0.3629	0.1892	0.5157

Table S3. Let-7 family associated with the development of HCC in the CHC patients with non-SVR after antiviral treatment (M6): Multivariate Cox proportional hazard regression model.

Og 10 2^-Delta Ct		Adjusted HR (95%CI)	*p*
Let7a	M6 median: ≥−1.88 vs <−1.88	0.58 (0.15–2.07)	0.4148
	Δ post(M6)-pre(baseline) median ≥0.18 vs <0.18	0.35 (0.07–1.23)	0.1069
Let7b	M6 median: ≥−1.44 vs <−1.44	1.12 (0.33–3.71)	0.8513
	Δ post(M6)-pre(baseline) median ≥0.14 vs <0.14	1.20 (0.37–3.74)	0.7545
Let7c	M6 median: ≥−2.02 vs <−2.02	0.79 (0.23–2.44)	0.6897
	Δ post(M6)-pre(baseline) median: ≥0.198 vs <0.198	1.20 (0.35–4.07)	0.7639
Let7d	M6 median: ≥−1.90 vs <−1.90	0.28 (0.05–1.11)	0.0709
	Δ post(M6)-pre(baseline) median: ≥0.170 vs <0.170	0.88 (0.26–2.82)	0.8367
Let7e	M6 median: ≥−1.67 vs <−1.67	0.78 (0.20–2.80)	0.7100
	Δ post(M6)-pre(baseline) median ≥0.150 vs <0.150	0.99 (0.24–3.49)	0.9885
Let7f	M6 median: ≥−2.16 vs <−2.16	0.40 (0.17–1.21)	0.1083
	Δ post(M6)-pre(baseline) median: ≥0.0918 vs <0.0918	0.83 (0.24–2.70)	0.7557
Let7g	M6 median: ≥−1.91 vs <−1.91	0.48 (0.12–1.69)	0.2580
	Δ post(M6)-pre(baseline) median: ≥0.12 vs <0.12	0.50 (0.11–1.59)	0.2560
Let7i	M6 median: ≥−1.696 vs <−1.696	0.09(0.01–0.45)	0.0022 *
	Δ post(M6)-pre(baseline) median: ≥0.177 vs <0.177	0.45 (0.10–1.55)	0.2153
miR-98	M6 median: ≥−2.235 vs <−2.235	0.51(0.16–1.51)	0.2234
	Δ post(M6)-pre(baseline) median: ≥0.069 vs <0.069	0.67 (0.19–2.16)	0.5017
